# Spatial distribution of active compounds in stratum corneum—partitioning between corneocytes and lipid matrix

**DOI:** 10.1038/s41598-024-66418-x

**Published:** 2024-08-12

**Authors:** Peter Sjövall, Sebastien Gregoire, William Wargniez, Lisa Skedung, Ann Detroyer, Gustavo S. Luengo

**Affiliations:** 1 RISE Research Institutes of Sweden, Borås, Sweden; 2 L’Oréal Research and Innovation, Aulnay-sous-Bois, France; 3 RISE Research Institutes of Sweden, Stockholm, Sweden

**Keywords:** Structural biology, Permeation and transport, Imaging studies, Mass spectrometry

## Abstract

The interaction of active substances with molecular structures in stratum corneum (SC) is crucial for the efficacy and safety of cosmetic formulations and topical drugs. However, the molecular architecture of SC is highly complex and methods to unambiguously localize exogenous molecules within SC are lacking. Consequently, little is known about the distribution of actives within SC, and proposed penetration mechanisms through SC are typically limited to simple diffusion via a tortuous (lipid only) or transverse (across corneocytes and lipid matrix) pathway. In this work, 3D mass spectrometry imaging is used to determine the spatial distributions of four active substances at subcellular resolution in SC, including partitioning between the corneocytes and the intercellular lipid matrix. The results indicate that caffeine, 2-methyl resorcinol and oxybenzone are homogeneously distributed in the corneocytes but largely absent in the lipid matrix, despite considerable differences in lipophilicity. In contrast, the distribution- of jasmonic acid derivative is more inhomogeneous and indicates considerable localization to both the lipid phase and the corneocytes.

## Introduction

Stratum corneum (SC) is the outermost layer of the skin and the main barrier between our inner bodies and the external environment. SC is also the target of many cosmetic ingredients, such as moisturizers, which influence the mechanical properties of the skin as well as a variety of sensorial aspects associated with aging^1^. In contrast to topical drugs, which may rely on penetration of the active therapeutical ingredient (API) through SC to reach their targets, the active ingredients of cosmetic formulations are designed to enter and remain in SC, in order to modify skin properties without risking systemic exposure. Understanding the distribution of exogenous molecules within SC is thus key to the development of safe and effective cosmetics and topical drugs, but also, e.g., to the ability to assess health hazards associated with exposure to environmental chemicals. However, detailed knowledge of molecular interactions of exogenous molecules in SC is very limited, mainly because of the complex molecular structure of SC and the lack of analytical methods capable of localizing specific molecules at sufficient spatial resolution in SC^2^.

In a design that keeps water inside and foreign substances outside our bodies, the SC consists of layers of flat corneocyte cells embedded in an extracellular lipid matrix; a structure commonly referred to as the “brick-and-mortar” model^3–6^. The unique composition of the lipid matrix (the mortar), containing approximately equimolar proportions of ceramides, cholesterol and free fatty acids, and the structural organization of the lipids in well-defined, stacked bilayers^6–8^ separating the corneocytes^7,8^ are critical for the barrier function of SC. For example, distortions in the lipid composition and/or bilayer structure have been shown to be associated with various skin disorders^5,6,9,10^. The corneocytes (the bricks) are keratin-rich, dead cells formed as the final stage of keratinocyte differentiation in viable epidermis^11–13^. Instead of a phospholipid cell membrane, the corneocytes have an outer shell of densely cross-linked proteins called the cornified envelope (CE), onto which a protein layer with a covalently bound monolayer of lipids (corneocyte lipid envelope, CLE) is facing the extracellular lipid matrix^14,15^. In addition to keratin fibers, the corneocyte interior contains filaggrin and so called natural moistering factors (NMFs), i.e., a mixture of small polar molecules that are important for the prevention of severe drying of SC^16^. Thus, the SC is a highly inhomogeneous structure, containing both hydrophilic and lipophilic domains, which an exogenous molecule may be exposed to during penetration into/through the SC.

The effective barrier properties of SC challenge our capability to deliver active cosmetic compounds or drug molecules into or through the skin, respectively. Models based on permeation data for a large number of compounds have shown that the major properties that determine the rate of permeation are the molecular weight (MW), where MW <  ~ 500 Da is a rough upper limit, and the lipophilicity expressed as log *P*, the logarithm of the octanol–water partition coefficient, where an intermediate value (log *P* ~ 0–5) has been found advantageous^2,17^. Molecular mechanisms to describe penetration through SC are frequently discussed in terms of a tortuous pathway, in which the molecules avoid the hydrophilic corneocytes and diffuse exclusively within the extracellular lipid matrix, and the transverse pathway, crossing the corneocytes, where more lipophilic molecules are expected to follow the tortuous pathway. Recent simulation studies indicate that other morphological parameters of the brick and mortar structure of the SC may also play a role^18^, including, e.g., skin hydration and permeability of the CE and CLE^16^. However, direct experimental support for either of these mechanisms have been difficult to obtain due to lack of experimental methods capable of providing unambiguous evidence for the spatial distribution of exogenous molecules within the SC^2,19,20^.

Time-of-flight secondary ion mass spectrometry (ToF–SIMS) is a mass spectrometry-based surface analysis method capable of molecular imaging at submicrometer lateral resolutions^21–24^. Furthermore, 3D distributions of specific molecules can be determined by combining 2D ToF–SIMS imaging with sputter erosion of the sample surface using Ar gas cluster ions (${\text{Ar}}_{{\text{n}}}^{ + }$, n = 1000–4000)^25–27^. Specifically, this type of cluster ions can be used to remove material from organic surfaces in a highly controlled and homogeneous manner without significant molecular damage at the remaining surface, allowing for molecular 3D ToF–SIMS analysis at a depth resolution in the range of 5–10 nm^26^. Recently, 2D and 3D ToF–SIMS have been increasingly used for characterization of skin, providing spatial distributions of topically applied compounds as well as endogenous lipid and protein components^28–36^. In this work, 3D ToF–SIMS is used to determine the spatial distributions of four actives with varying lipophilic properties (log *P* from − 0.07 to 3.79) in tape strip SC samples, prepared from human ex vivo skin treated with topical formulations containing mixtures of these actives. We show that caffeine, 2-methyl resorcinol (2-MR) and oxybenzone are primarily localized to the corneocytes, whereas jasmonic acid derivative (JAD, also known as LR2412) is located both to the corneocyte bodies and the lipid matrix. For all four actives, the results suggest a largely transverse penetration pathway and in no case a purely tortuous pathway.

## Results

### 3D distribution of endogenous skin components

**Figure 1:**
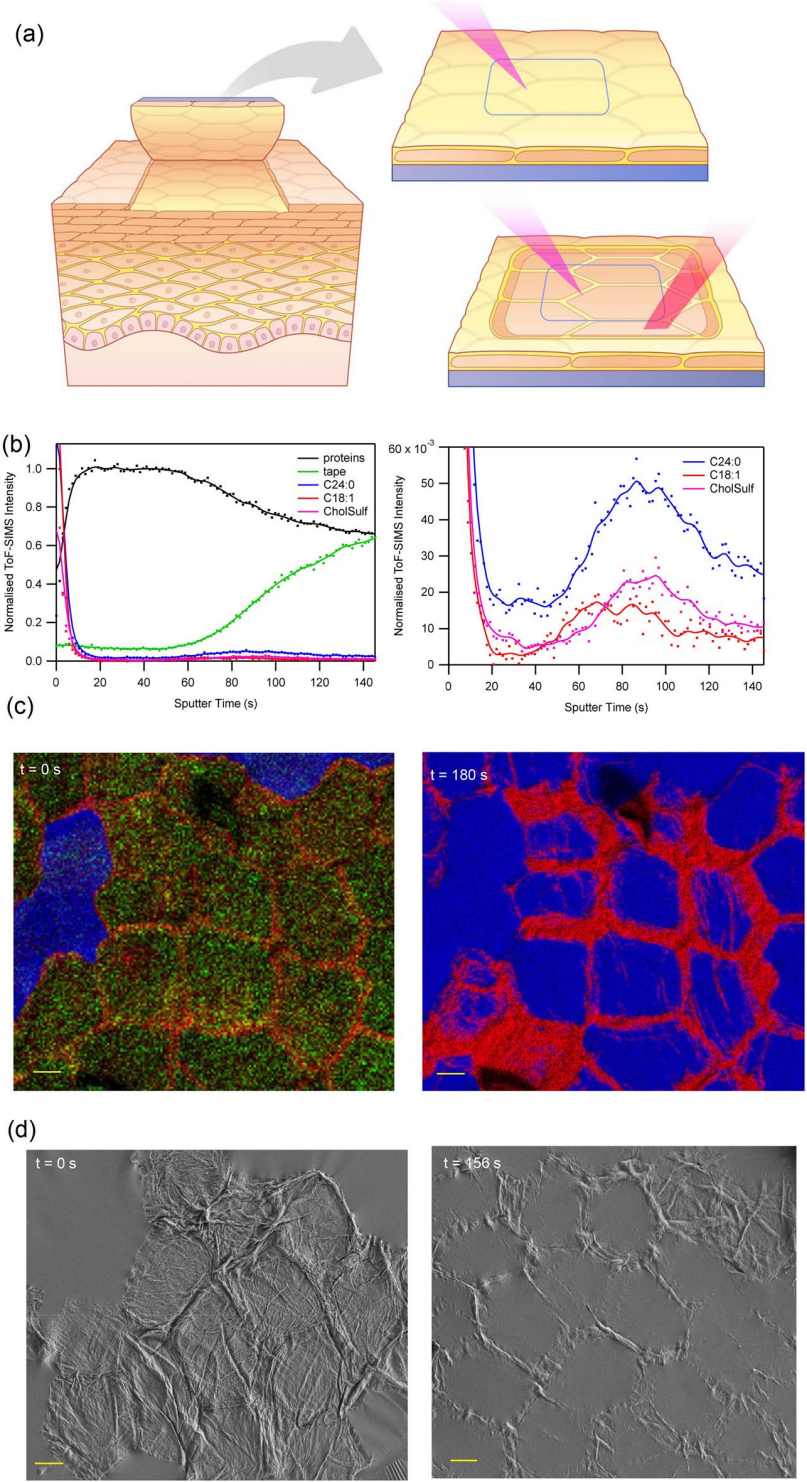
3D ToF–SIMS and SEM analysis of tape strip sample. (**a**) Schematic representation of the measurement principle, including tape stripping, ToF–SIMS imaging analysis of the top lipid surface and 3D analysis by combined 2D analysis and sputter etching. The analysis area is smaller than the sputter area to ensure homogeneous etching rates. (**b**) Depth profiles of ions representing proteins, tape and skin lipids (C24:0, C18:1 and cholesteryl sulfate). A sputter time of 82 s corresponds approximately to 10^15^ ions/cm^2^. (**c**) ToF–SIMS ion images of the tape strip sample prior to (t = 0 s) and after (t = 180 s) 3D ToF–SIMS analysis, showing proteins in red, tape in blue and skin lipids (C24:0 + C26:0) in green. (**d**) SEM micrographs of corneocytes on the tape surface prior to (t = 0) and after (t = 156 s) 3D ToF–SIMS analysis (different areas). Note the residual proteins at the corneocyte periphery after 3D ToF–SIMS analysis. See Table 1 for ions used to represent the different components in (**b**,**c**). Scale bars in (**c**,**d**) are 10 µm.

Spatial distributions of endogenous molecular components and active compounds were determined by 3D ToF–SIMS analysis of tape strip samples collected at different depths into SC (represented by consecutive tape strips acquired from the same skin area). Each tape strip sample comprise (ideally) a single corneocyte layer embedded in a thin layer of skin lipids, transferred onto a tape substrate by attaching and detaching the tape from a skin surface^37^ (Fig. 1a). Thus, initial ToF–SIMS analysis of the tape strip probes specifically the lipid matrix located on top of the corneocytes, due to the high surface specificity of this measurement (< 1 nm)^38^. Upon sputter erosion, the thin lipid layer is removed, allowing for analysis of the corneocyte bodies, followed by gradual removal of also the corneocyte bodies, thereby exposing the underlying lipid layer and tape substrate for analysis (Fig. 1a). Repeated 2D ToF–SIMS imaging measurements during the entire sputter erosion process provides 3D information (with the sample depth represented by sputter time), including (i) depth distributions of specific molecular species from depth profiles (Fig. 1b), (ii) 2D distributions of specific molecular species at different depths into the lipid/corneocyte layer, represented by ion images acquired after different sputter times (Fig. 1c), and (iii) molecular composition of separate lipid and corneocyte compartments, respectively, from mass spectra of selected 3D regions (Supplementary Fig. S1).

The different compartments of the tape strip sample are clearly discernible in the depth profiles (Fig. 1b), where the outer lipid phase is represented by initially high, but rapidly decreasing, intensities of the skin lipids (sputter time 0–5 s) and the underlying corneocyte region is represented by an extended, flat maximum of the protein intensity, accompanied by very low lipid intensities (20–46 s). At the end of the corneocyte region (at around 50 s), a simultaneous decrease in protein intensity and increase in tape intensity signals the start of a regime characterized by a gradually decreasing fraction of the sample surface being covered by corneocyte material, and an increasing fraction of the underlying tape substrate being exposed. Magnification of the vertical scale reveals maxima in the skin lipid profiles (Fig. 1b, right panel) at about 90–100 s for C24:0 and cholesteryl sulfate and at 60–70 s for C18:1, consistent with detection of the thin lipid matrix layer between the corneocytes and the tape substrate. The shifted maximum for C18:1, as compared to C24:0 and cholesteryl sulfate, is consistent with results of a previous study, in which the C18:1 distribution was found to be associated with cholesteryl oleate^32^. It should be pointed out, however, that it is not known whether these distributions are affected by the fact that ex vivo skin was used, which in contrast to in vivo skin is in equilibrium with regards to 3D molecular distributions.

ToF–SIMS ion images and scanning electron microscopy (SEM) micrographs acquired after 3D ToF–SIMS analysis (Fig. 1c,d, Supplementary Fig. S2) demonstrates the presence of protein residues on the tape surface, even after very long sputter times. The protein residues are particularly prominent at the periphery region of the corneocytes, whereas the tape is exposed at the corneocyte centers, indicating complete removal of the corneocyte material. In addition to the relatively thick protein residues at the corneocyte periphery, thinner protein fibers (possibly bundles of keratin fibers) are also observed, occasionally crossing the corneocyte centers (Supplementary Fig. S2). Given a constant and homogeneous sputter erosion rate across the analysis area, the protein residues can be assumed to reflect thicker regions of protein material in the deposited corneocytes on the tape strip sample. Indeed, SEM and ToF–SIMS images acquired prior to sputter erosion (Fig. 1c,d) demonstrate a variable topography of the corneocyte surfaces and elevated protein structures at the corneocyte edges. The strong topography of the corneocyte surfaces may be rationalized by the fact that the corneocytes are completely dehydrated and largely collapsed on the tape surface in the vacuum environment during analysis. Furthermore, the apparent protein accumulation at the corneocyte periphery is consistent with observations of elevated concentrations of corneodesmosomes at the corneocyte periphery, as well as possible morphological variations of the cornified envelope^12^. In the depth profiles, the inhomogeneous thickness of the corneocytes is reflected in the gradual changes of the protein and tape intensities after conclusion of the protein plateau at 90–100 s and the broad maxima of the lipid intensities representing the lipid layer between the corneocytes and the tape (Fig. 1b), as variable sputter times are needed to remove the corneocyte material and expose the underlying lipid matrix and tape, across the analysis area.

### 3D distribution of active compounds

**Figure 2:**
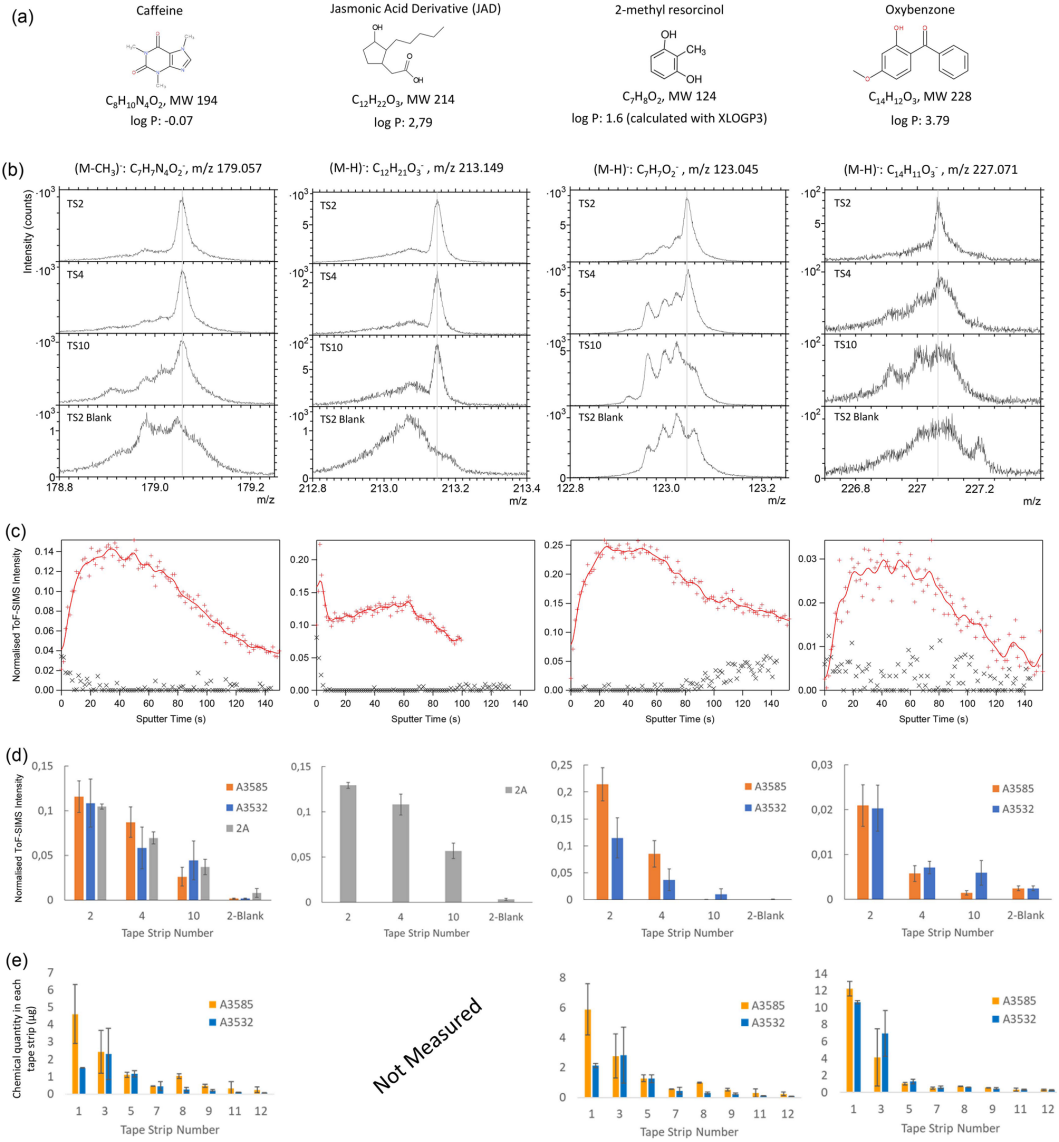
Spatial distributions of caffeine, JAD, 2-MR and oxybenzone in SC. (**a**) Selected properties of the four actives, (**b**) negative ToF–SIMS spectra of the ions used to monitor the four actives, from analyses of tape strips #2, #4, #10 and from tape strip #2 of a skin sample not treated with the actives (“Blank”). Note that the spectra are individually normalized to show the peak intensities of the actives in relation to the skin background. (**c**) Depth profiles of the four actives in tape strip #2 (red curves). Note the different depth profile for JAD, displaying a peak in the lipid matrix region, as compared to the other three actives, which show depth profiles with similar features as the protein. The black symbols show the background signal obtained from the “Blank” sample. (**d**) Relative quantification of actives concentration versus depth in SC, i.e. TS2, TS4 and TS10, as determined from the ToF–SIMS depth profiles. Displayed intensities are mean values and error bars correspond to +/− 1 standard deviation (N = 3). (**e**) Quantification of active concentrations as determined by LC–MS/MS for tape strips from the same skin sample as analysed by ToF–SIMS. Bars display mean values with ± 1 sd (samples in duplicate).

The four actives, caffeine, JAD, 2-MR and oxybenzone (Fig. 2a), were detected with high specificity in the tape strip samples using peaks corresponding to molecular ions (or, for caffeine, nearly molecular ions), which are clearly distinguished from the skin background in the mass spectra (Fig. 2b, Supplementary Fig. S3). The measured depth profiles of caffeine, 2-MR and oxybenzone revealed features that are very similar to the protein depth profiles, i.e., an initially increasing intensity to a maximum plateau followed by a gradual decrease, thus indicating localization primarily to the corneocytes for these actives (Fig. 2c). In contrast, JAD displayed a profile comprising a peak at the initial, lipid matrix-associated regime, followed by a flat or slowly increasing intensity in the corneocyte region and a final decrease corresponding to the gradual replacement of corneocyte residues with tape on the sample surface. Thus, the JAD profile provides clear evidence for localization of this active to both the lipid matrix and the corneocyte bodies.

Whereas the depth profiles in Fig. 2c were acquired from the second consecutive tape strip (TS2), similar shapes of the depth profiles were obtained from TS4 and TS10, indicating that the partitioning between lipid matrix and corneocytes remains essentially unchanged with increasing depths into SC for all four actives (Supplementary Fig. S4). Furthermore, the relative concentrations of the active compounds were determined from the depth profiles of TS2, TS4 and TS10 by normalizing the accumulated signal intensities of the actives to those of the proteins (see “Methods” section), providing estimates of the depth distributions into SC. For all four actives, the results show considerable concentration reductions with increasing depth into SC (Fig. 2d). However, the depth distributions are less steep for caffeine and JAD, with smaller relative decrease between TS2 and TS4, as compared to 2-MR and oxybenzone. For verification and quantification, the concentrations of caffeine, 2-MR and oxybenzone in the intermediate tape strips from the same skin sample were determined by liquid chromatography coupled with tandem mass spectrometry (LC–MS/MS) (Fig. 2e). The results are in good agreement with the depth distributions obtained from the ToF–SIMS data (Fig. 2d) and, in addition, consistent with a previous study showing the classical decreasing profile for JAD as a function of depth in the SC (despite a different vehicle was used compared to the present work)^19^.

**Figure 3:**
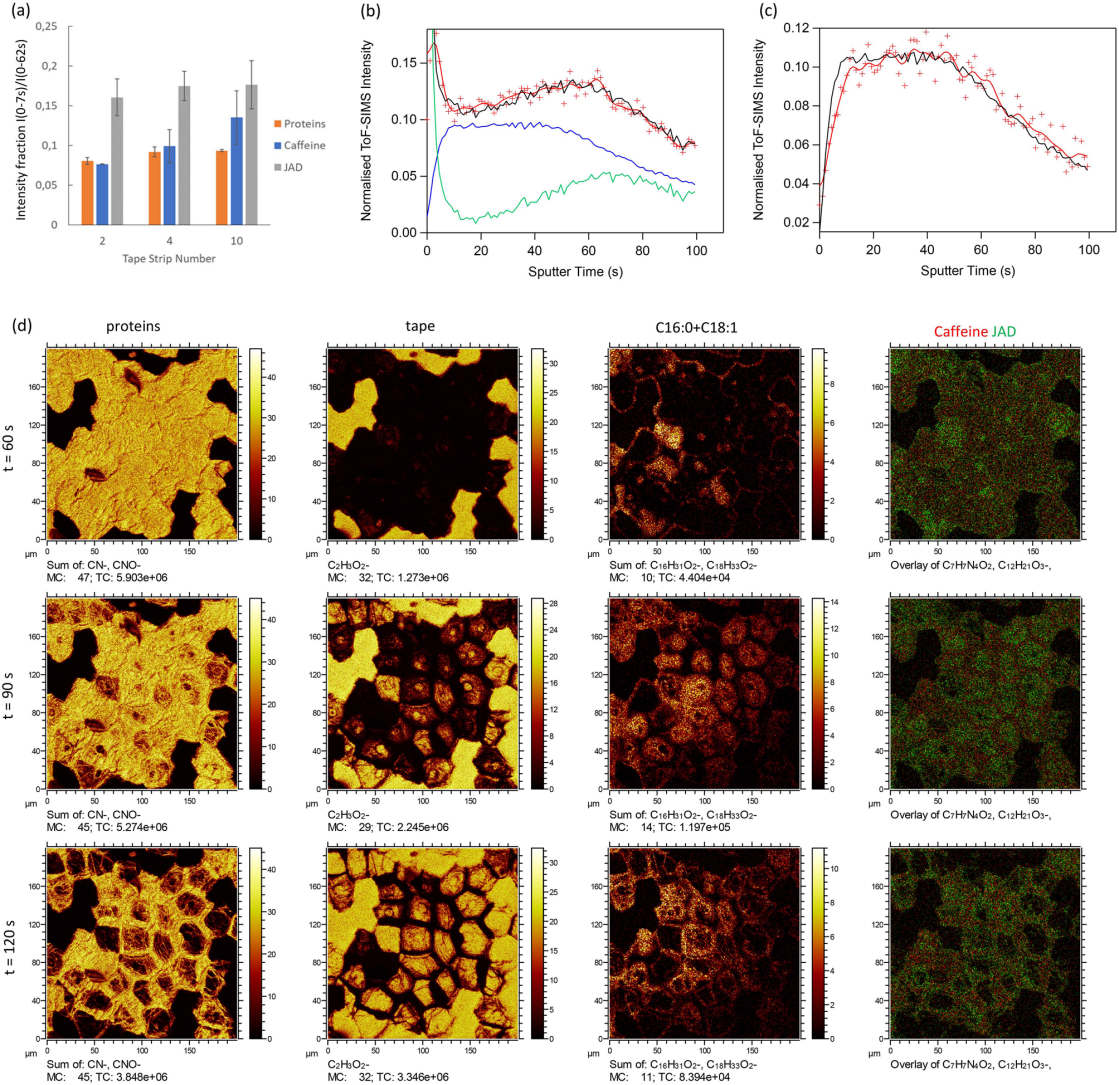
3D distributions of caffeine and JAD in lipid matrix versus corneocytes. (**a**) Fraction of actives detected in the top lipid region relative to the combined top lipid and corneocyte regions, estimated from the depth profiles as the ratio of measured intensities in the lipid region (t = 0–7 s) to total intensities in the lipid and corneocyte region (t = 0–62 s). The protein results represent the expected ratio for corneocyte localization, whereas higher values correspond to increasing fractional localization to the lipid matrix. Mean values and error bars corresponding to +/− 1 standard deviation (N = 3). (**b**) Depth profiles (from TS2) of JAD (red), protein (blue) and C18:1 (green), together with a fit to the JAD profile (black) given by a weighted sum of the protein profile and C18:1. (**c**) Depth profile of caffeine (same measurement as in (**b**)), where the black line is a fit using the protein depth profile only. (**d**) Ion images acquired from tape strip sample (TS2) after t = 60, 90 and 120 s of sputter erosion, showing proteins, tape, C18:1 and overlay images of caffeine (red) and JAD (green). Note the correlation between the distributions of C18:1 and JAD, whereas the distribution of caffeine is homogeneous across the protein-covered parts of the surface.

A quantitative representation of the partitioning between lipid matrix and corneocytes was obtained for caffeine and JAD from the intensity ratio of the detected signal in the top lipid region of the depth profile (0–7 s) to that in the top layer also including the corneocyte region (0–62 s), see Fig. 3a. Here, the intensity ratio of the protein depth profiles serves as a reference corresponding to complete localization to the corneocyte, whereas higher values represent increasing fractions of the active being localized to the lipid matrix. Due to possible matrix effects, however, the intensity ratios between lipid matrix and corneocytes cannot be considered as quantitative concentration ratios, but they can be used to compare different samples/measurements. Roughly constant values of this “lipid phase fraction” were observed for JAD in TS2, TS4 and TS10, at about twice the values for proteins, thus confirming significant JAD localization to both lipid matrix and corneocytes and that the lipid/corneocyte partitioning is roughly unchanged as a function of depth into SC. For caffeine, the fraction that is detected in the lipid region of the depth profile is similar to that for proteins, consistent with localization mainly to the corneocytes. A possible trend towards increasing localization to the lipid region with increasing depth into SC is apparent for caffeine, but the significance of this trend is unclear.

A correlation was observed between JAD and the C18:1 fatty acid, in both the depth profiles and 2D ion images (Fig. 3b–d, Supplementary Figs. S5, S6), indicating a spatial association between these components in the skin structure. Firstly, it was found that the JAD depth profile in several cases could be largely reproduced by a weighted sum of the protein and C18:1 depth profiles (Fig. 3b), thus indicating a correlation of JAD with both C18:1 and proteins in the depth direction. In contrast, the caffeine profile fits well with the protein profile only (Fig. 3c). Secondly, ion images acquired in the depth region around the bottom of the corneocyte layer show the appearance of a thin lipid layer containing C18:1 between the corneocytes and the tape substrate and, furthermore, that elevated intensities of JAD are colocalized with C18:1 in this layer (Fig. 3d, Supplementary Figs. S5, S6). In contrast, caffeine displays a homogeneous distribution in areas with remaining corneocyte residues, i.e., areas with high protein intensities, consistent with localization mainly to the corneocytes. For JAD, the elevated intensities associated to C18:1 are superimposed on a homogeneous distribution in areas with remaining proteins/corneocytes (particularly evident in Supplementary Fig. S6), as expected for JAD localization to both the lipid matrix and corneocytes.

### Morphological details of the corneocyte structure

Detailed inspection of ion images from the corneocyte/tape interface reveals additional details of the corneocyte structure and morphology (Fig. 3d, Supplementary Figs. S2, S5 and S6). At a sputter time of 60 s, the protein image is bright and largely homogeneous in the area covered by corneocytes, whereas the tape image is dark in this area, indicating that corneocyte material still covers the surface and that the sputter erosion has not yet reached the tape substrate. At 90 s, the underlying tape surface has partially appeared at the center of most corneocytes, accompanied by correspondingly darker areas in the protein image, indicating partial removal of the corneocyte material from the cell centers. At 120 s of sputter erosion, more of the proteins have been removed and more of the tape substrate is exposed at the centers of the corneocytes. However, the corneocyte periphery areas are still covered by protein residues, and thin fibers (about 1–2 µm diameter) crossing the corneocyte centers are clearly visible in the protein image. The presence of a thin C18:1-containing lipid layer below the corneocytes is evident in the C18:1 images, showing the appearance of bright areas at the corneocyte centers in parallel with corresponding dark and bright areas in the protein and tape images, respectively. With increasing sputter erosion, also the lipid layer is gradually removed, leaving the tape increasingly exposed on the surface. Furthermore, in the ion images acquired after 90 s sputter erosion, the presence of subcircular structures, one in each corneocyte is evident (Fig. 3d). The origin of these structures is as yet unknown, but the increased tape signal (and decreased protein signal) in these structures at t = 90 s indicate a reduced protein thickness, possibly caused by an elevated water content of this structure in the hydrated state.

### Corneocyte double layers

**Figure 4:**
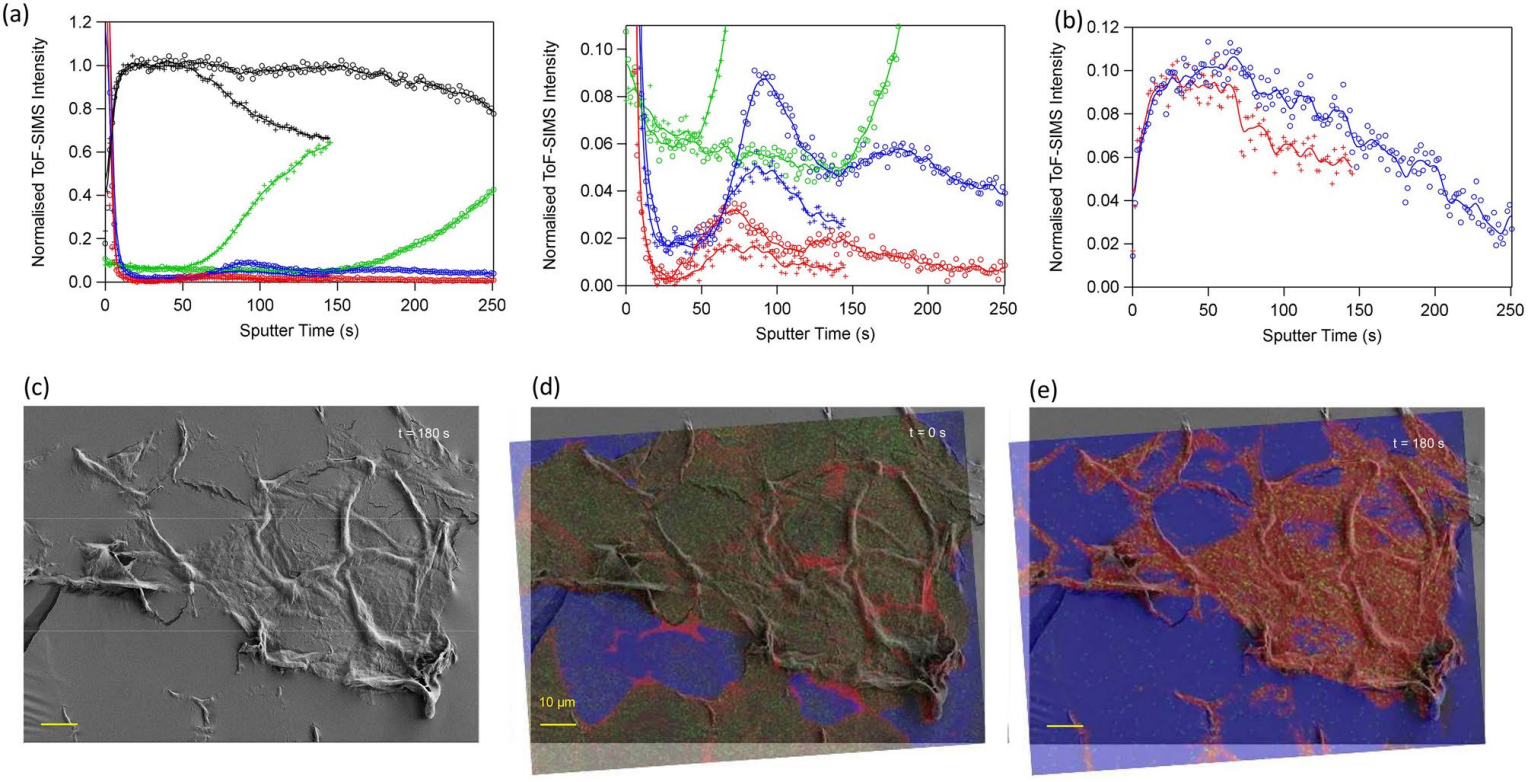
ToF–SIMS data of double corneocyte layer in tape strip sample. (**a**) Depth profiles of proteins (black), tape (green), C24:0 (blue) and C18:1 (red) from two measurements of the same tape strip sample, attributed to a single corneocyte layer (crosses) and a double corneocyte layer (circles). Note the matching intensity maxima of the two C24:0 profiles at t ≈ 95 s, and the presence of another maximum for the double layer profile at approximately twice the sputter time, t ≈ 180 s. (**b**) Caffeine depth profiles of the same measurements as in (**a**), attributed to single (red crosses) and double (blue circles) corneocyte layers. (**c**) SEM micrograph of tape strip sample after 180 s sputter erosion, i.e., well over the sputter time needed to remove most of a single corneocyte layer. (**d**,**e**) SEM image as in (**c**) with superimposed ToF–SIMS images, showing proteins in red, tape in blue and SC lipids (C24:0 + C26:0) in green, of the same area (**d**) prior to sputter erosion (t = 0 s), and (**e**) after sputter erosion (t = 180 s).

In most cases, the tape strip samples comprised a single corneocyte layer transferred onto the tape substrate. However, the 3D ToF–SIMS analysis occasionally revealed evidence for double corneocyte layers deposited on the tape substrate (Fig. 4). Firstly, the corneocyte region in the protein depth profiles (i.e., the constant plateau) is extended to about twice the sputter time for a single corneocyte layer, with a corresponding delayed onset of the increase in tape intensity (Fig. 4a). Given a constant sputter erosion rate, these depth profiles are consistent with sputter erosion through two consecutive corneocyte layers. In addition, the C24:0 lipid profile shows a clear maximum at the same sputter time as for a single corneocyte layer, i.e., at a sample depth corresponding to a lipid layer between two corneocyte layers, and then another (weaker) maximum at approximately twice the sputter time of the first, representing the lipid layer below the bottom corneocyte layer. The second C24:0 maximum is preceded by the onset of the increasing tape intensity, thus consistent with the lipid layer between the second corneocyte layer and the tape substrate. The C18:1 profile similarly shows two maxima, which both are equally shifted to shorter sputter times, consistent with previous results^32^. The caffeine profile surprisingly indicates a gradually decreasing concentration upon sputter erosion into and through the second corneocyte layer (Fig. 4b); an observation that, however, requires further verification and investigation.

The occasional double corneocyte layers can also be observed in ion images acquired after increasing sputter erosion times (Fig. 4e, Supplementary Fig. S7). Superimposed SEM and ion images acquired after 180 s sputter erosion, i.e. long after removal of the first corneocyte layer and just at the onset of tape exposure below the second corneocyte layer (according to the depth profile in Fig. 4a), shows a distinct area with largely continuous protein residues (only minor tape spots exposed), whereas the adjacent area is mainly bare tape and isolated corneocyte edge structures (Fig. 4c,e). Ion images of the same analysis area acquired prior to start of the sputter erosion show the presence of lipid-covered corneocytes over a large part of the tape strip surface (Fig. 4d), thus indicating that the area with remaining protein residues after 180 s sputter erosion corresponds to a double corneocyte layer, whereas part of the adjacent area with mainly bare tape was originally covered by a single corneocyte layer. These observations indicate that the “ideal” view of tape stripping as a layer-by-layer process may not always be correct, with important consequences for quantification measurements based on this sampling method. Usually, quantification of the amount of SC removed by tape stripping is done by double weighing^39^ or alternatively by contrast image method^40^. In the absence of such measurements, the amount of SC removed by tape stripping is unknown and the resulting distribution profile within SC has to be considered with attention.

## Discussion

**Figure 5:**
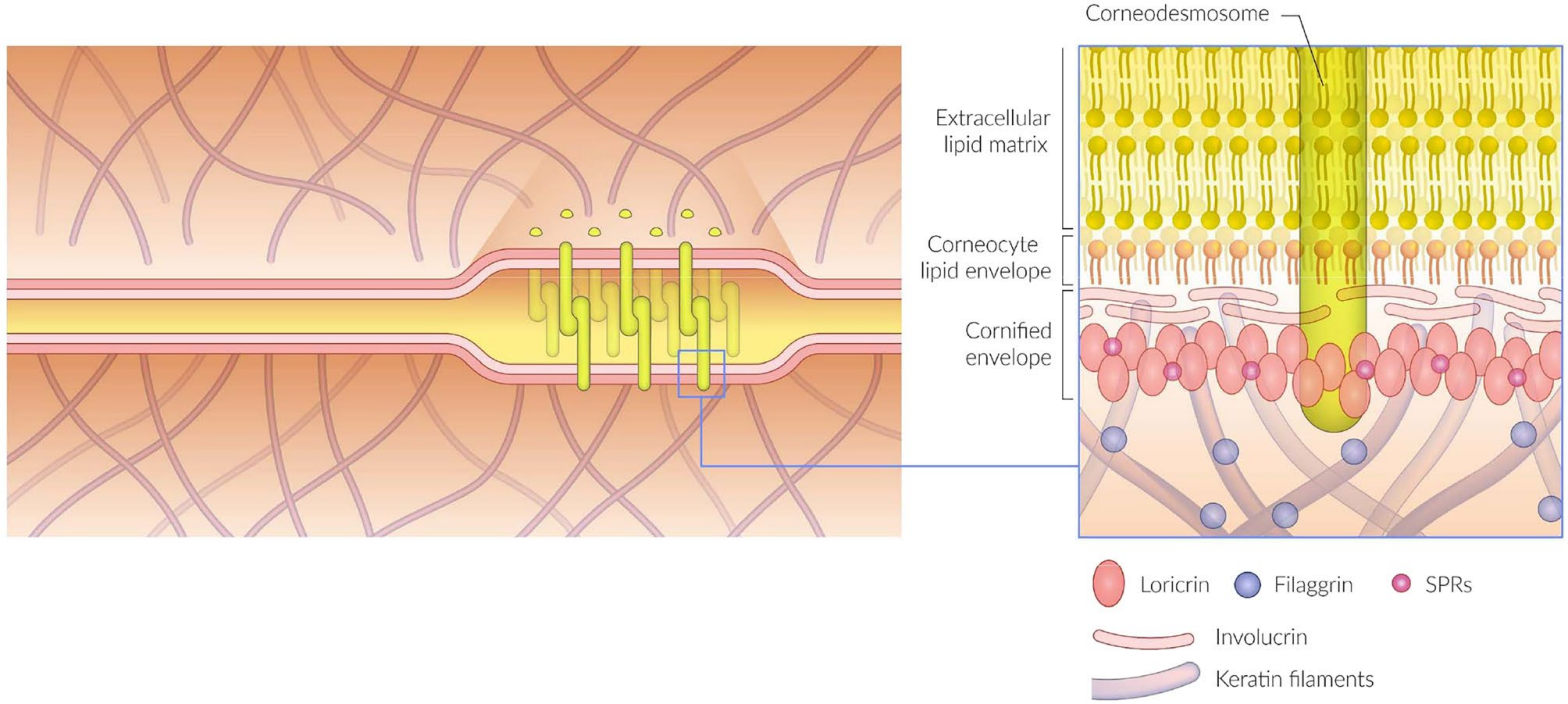
Schematic image of the interface region between the corneocytes and the extracellular lipid matrix of SC, including the cornified envelope and the corneocyte lipid envelope, adapted from Egawa and Kabashima^43^.

The observation that caffeine, 2-MR and oxybenzone are primarily localised to the corneocytes indicates that the varying lipophilic properties of these compounds do not have a critical effect on their partitioning between corneocytes and lipid matrix in SC. This result is quite different from that pointed out in previous studies^16,19,20^ and may seem unexpected when considering a simple brick–mortar model, which describes the SC structure as hydrophilic corneocytes embedded in a lipophilic matrix. However, the well-ordered bilayer structure of the lipid matrix^7,41^, critical for its barrier properties, and the complex morphological and molecular structure of the corneocytes^12^ (schematically shown in Fig. 5) may suggest that the lipid/corneocyte partitioning is determined by more complex factors and cannot be explained as a simple dependence on log *P*. In fact, there is little experimental evidence for a simple correlation between lipophilicity and lipid/corneocyte partitioning. On the contrary, it was recently shown that the lipid/corneocyte partitioning is very similar for caffeine and 2-MR, despite a considerable difference in lipophilic properties^42^, in good agreement with the results of the present study.

Specifically, our results demonstrate that JAD is the only of the four actives with a significant localisation in the lipid matrix, despite the fact that oxybenzone is a more lipophilic molecule, according to the log *P* values (2.79 and 3.79, respectively), and that their molecular weights are similar (214 and 228 Da, respectively). A possible explanation for the enhanced affinity of JAD to the lipid matrix may instead be related to the specific molecular structure of JAD compared to the other actives, namely that JAD is the only structure with an aliphatic chain, and also an ionizable group and surfactant amphiphilic properties. We speculate that the aliphatic portion and/or either of the other two of these properties may be advantageous for incorporation (anchorage) into the lipid bilayer structure.

As the most hydrophilic of the four actives, caffeine was a good candidate for high affinity and preferential localization to the corneocytes, which was also observed. More surprising, perhaps, were the observations that also the other three, considerably more lipophilic actives displayed strong affinities to the corneocytes. However, these observations are consistent with previous studies suggesting highly complex, and not entirely hydrophilic properties of the keratin fiber surfaces in the corneocytes^11,44^. For example, based on confocal Raman results, Choe et al.^11^ suggested a three-layer model, in which the surfaces of the keratin fibers in the upper 30% of the corneocyte layers of SC are relatively hydrophobic with reduced capacity to bind water, whereas those in the deeper corneocyte layers are more hydrophilic, making them more prone to bind water and induce corneocyte swelling. This model is consistent with our results as it provides potential binding sites for also relatively lipophilic molecules inside the corneocytes, to explain the localization of the actives to the corneocytes, and also the localization of these sites to the upper corneocyte layers, to explain the observed steep depth distributions into the SC, especially for 2-MR and oxybenzone. In the case of JAD, it may be argued that the three-layer model would generate a varying lipid-corneocyte partitioning with increasing depth into SC, as the capacity to bind JAD inside the corneocytes would be reduced. However, other possibilities to incorporate the lipophilic compounds inside the corneocytes may exist, e.g., including mechanisms involving the surfactant properties of JAD.

The results showed clear evidence for spatial colocalization of JAD with C18:1 fatty acid in the tape strip samples. Furthermore, C18:1 was found to be spatially separated from the “typical” skin lipids, here represented by C24:0, C26:0 and cholesteryl sulfate, with more inhomogeneous 2D distributions in the top lipid layer and depth distributions of the bottom lipid layer shifted slightly upwards, towards the sample surface. Similar observations were made in a previous study of SC patches containing multiple corneocyte layers, i.e., inhomogeneous C18:1 2D distributions and shifted depth profiles compared to the “typical” skin lipids^32^. In that previous study, the C18:1 signal was clearly shown to represent cholesteryl oleate in the corneocyte layer structure. We therefore speculate that the C18:1 signal also in the present study represent cholesteryl oleate and that it is spatially separated from the intercellular matrix, possibly located to the cornified envelope or the cornified lipid envelope (Fig. 5).

A low concentration of the active in the lipid matrix is consistent with a penetration mechanism in which crossing the lipid barrier is the limiting factor, in good agreement with the well-established importance of the morphological and molecular integrity of the lipid matrix for the barrier function of SC^5,10,45^. However, even at low concentrations in the lipid matrix, the penetration would be expected to be sensitive to the lipophilic properties of the active compound, consistent with permeation data^2^, as the lipophilicity may strongly affect the rate by which the active can cross the lipid barrier. While strongly suggesting that penetration through the corneocytes is not a limiting factor, our results showing low concentrations of caffeine, 2-MR and oxybenzone in the lipid matrix thus suggest that local effects may be important for the penetration, such as penetration at defects and/or at interfaces of adjoining regions/phases in the lipid bilayer structures, as previously discussed^16^.

The 3D ToF–SIMS technique used in this work offers novel opportunities to study corneocyte morphology, as, e.g., demonstrated by the observation of protein residues in distinctly defined regions at the corneocyte periphery (Fig. 1c,d). While these observations clearly indicate thicker and/or more compact protein structures at the periphery of the corneocytes, as compared to the centers, the exact origin of these residues remains unclear and may warrant further investigation. Interestingly, similar spatial distributions observed for caffeine absorption into skin by coherent anti-Stokes Raman spectroscopy (CARS) imaging led the authors to conclude that caffeine is primarily localized to the lipid phase^19^, assuming that the lipid phase is localized at the corneocyte edges. However, the results of the present study instead suggest increased caffeine abundance at the corneocyte edges due to protein accumulation in combination with caffeine localization to proteins inside the corneocytes. This interpretation is also more consistent with the observed thickness of the caffeine edge structures by CARS (around 5 µm), as opposed to the expected dimension of the lipid layer between corneocytes in the same layer, i.e. around 100–200 nm.

Although the results of the present study are quite clear, questions remain as to whether the observed partitioning and 3D distributions may have been affected by the exact conditions under which the samples were prepared and analysed, e.g., regarding skin hydration. While the conditions during application of the formulations on the skin samples were very well controlled, corresponding to infinite dose of the vehicle for sufficient time to reach steady-state (4 h) under occlusive conditions, it is not clear to what degree the hydration was retained during tape stripping and subsequent storage (at − 20 °C and − 80 °C). During 3D ToF–SIMS measurements, the tape strip samples were clearly completely dehydrated, as the analyses were carried out with the samples in vacuum at room temperature. Results of the type presented in this study will potentially contribute to a more detailed understanding of the absorption and diffusion of topically applied molecules in SC, e.g., in combination with in silico modelling studies. Given the novelty of the presented methodology and the results, several important follow-up experiments can be considered, e.g., (i) 3D ToF–SIMS analyses under cryogenic sample conditions (< 150 °C), which would retain the hydration of the tape strip samples and make possible the mapping of water 3D distributions, together with those of the other endogenous and exogenous components, (ii) studies of additional compounds, in particular such with higher log *P* values and/or other molecular properties that can be expected to favor high partitioning in the lipid matrix, (iii) studies of the effect of vehicle on the penetration and 3D distribution of active compounds, including the effect of penetration enhancers.

## Methods

### Study outline

The study includes analyses of tape strips from human ex vivo skin samples treated with two different formulations: one containing a mixture of caffeine and JAD, and one with a mixture of caffeine, 2-MR and oxybenzone. Each of the two formulations were treated to skin from two different donors (i.e., four donors in total) and 12 consecutive tape strip samples (TS1–TS12) were prepared from each of these skin samples, as well as from untreated skin samples from the same donors.

For each of the four donors, 3D ToF–SIMS analysis was carried out on TS2, TS4, and TS10 from the treated skin samples, providing a selection of increasing depths into SC but excluding TS1 to avoid possible artefacts at the skin surface, and TS2 from the untreated skin samples. For each of the tape strip samples, the 3D ToF–SIMS analysis included (i) 3D measurements of negative ions at three different locations, and of positive ions at one location, with the instrument optimized for high mass resolution, (ii) 3D measurements of negative ions at one location with the instrument optimized for high image resolution, (iii) acquisition of 2D high-resolution images of negative ions after increasing sputter erosion times, typically 0, 30, 60, 90, 120 and 180 s.

Absolute quantification and depth distribution of caffeine, 2-MR and oxybenzone in SC was obtained by LC–MS/MS analysis on all tape strip samples that were not analyzed by 3D ToF–SIMS.

In addition, tape strip samples from three of the donors were analyzed by SEM in areas subjected to 3D ToF–SIMS analysis, i.e., areas where the corneocyte had been largely removed by sputter erosion, as well as in areas with intact corneocytes not subjected to sputter erosion. Occasionally, areas exposed to sputter erosion for times shorter than the typical total sputter times from the 3D measurements were also analyzed by SEM.

### 3D ToF–SIMS

3D ToF–SIMS analyses were carried out on a TOFSIMS 5 instrument (IONTOF GmbH) using 30 keV ${\text{Bi}}_{3}^{ + }$ primary ions for analysis and 10 keV ${\text{Ar}}_{{{2}000}}^{ + }$ ions for sputter erosion. Negative and positive ion data were acquired with the instrument optimized for high mass resolution (bunched mode, m/Δm = ca 6500) or for high image resolution (delayed extraction mode (DE), m/Δm = ca 2500) with low-energy electron flooding for charge compensation. 3D measurements were performed by alternating cycles of 2D analysis (${\text{Bi}}_{3}^{ + }$: analysis area 200 × 200 µm^2^, pulsed current 0.18 and 0.055 pA for bunched and DE modes, respectively, analysis time per cycle 13.11 and 39.1 s for bunched and DE modes, respectively) and sputter erosion (${\text{Ar}}_{{{2}000}}^{ + }$: sputter area 600 × 600 µm^2^, current 6–8 nA, sputter time per cycle 1.47 and 2.94 s for bunched and DE modes, respectively).

The 3D ToF–SIMS data was evaluated using the SurfaceLab 7.3 (IONTOF GmbH) and Igor Pro 9 (Wavemetrics, Inc.) softwares. Ions used to represent the active compounds were selected from reference spectra acquired for the pure substances (Supplementary Fig. S3), as listed in Table 1 together with ions used to represent the endogenous skin components. Due to detector saturation for the CN^-^ and ${\text{C}}_{{2}} {\text{H}}_{{3}} {\text{O}}_{2}^{ - }$ ions representing proteins and tape, respectively, less abundant isotopes were used to monitor these components in the bunched mode. Depth profiles were generated from regions of interest (ROIs) displaying apparently homogenous corneocyte layers within the analysis areas, and using background-subtracted, Poisson-corrected intensities of the selected ions. The depth profiles were normalized to the protein intensity at the plateau of the depth profiles (corneocyte region), thus allowing for quantitative comparison of relative concentrations between measurements/samples. Relative quantification of the total amount of actives in the samples was done by summing/integrating the normalized intensities (using Excel, Microsoft, Inc.) from start of the depth profile to the end of the corneocyte region (sputter time t_sp_ = 0–62 s). Furthermore, the fraction of the active present in the lipid matrix, relative to the total amount, was estimated by calculating the ratio between the integrated intensity in the lipid region (t_sp_ = 0–7 s) and the total integrated intensity also including the corneocyte region (t_sp_ = 0–62 s). Assuming that the protein intensity comes exclusively from corneocytes, the ratios obtained for the protein depth profiles correspond to localization exclusively to corneocytes, whereas higher ratios indicate increasingly higher fractions of the active being present in the lipid matrix. All the presented results for depth profiles and mass spectra were obtained for negative ions and in the bunched mode, whereas the ion images were acquired in DE mode.

**Table 1:** List of negative ions used to represent active compounds and endogenous skin components in the evaluation of the acquired ToF–SIMS data.

Ion	m/z (measured)	Theoretical mass (u)	Component	Comment
CN^−^	26.005	26.003	Proteins	DE mode
^13^CN^−^	27.007	27.006	Proteins	Bunched mode
CNO^−^	42.003	41.998	Proteins	DE mode
C_2_H_3_O_2_^−^	59.018	59.013	Tape	DE mode
^13^CCH_3_O_2_^−^	60.018	60.017	Tape	Bunched mode
C_16_H_31_O_2_^−^	255.230	255.232	C16:0 fatty acid	CE or TAG fragment
C_18_H_33_O_2_^−^	281.246	281.248	C18:1 fatty acid	CE or TAG fragment
C_24_H_47_O_2_^−^	367.356	367.358	C24:0 fatty acid	(M-H)^−^/CER fragment
C_26_H_51_O_2_^−^	395.386	395.389	C26:0 fatty acid	(M-H)^−^/CER fragment
C_27_H_45_SO_4_^−^	465.303	465.304	Cholesteryl sulfate	(M-H)^−^
C_7_H_7_N_4_O_2_^−^	179.056	179.057	Caffeine	(M-CH_3_)^−^
C_12_H_21_O_3_^−^	213.148	213.149	JAD	(M-H)^−^
C_7_H_7_O_2_^−^	123.045	123.045	2-MR	(M-H)^−^
C_14_H_11_O_3_^−^	227.070	227.071	Oxybenzone	(M-H)^−^

### Skin samples preparation

Abdominal human skins were obtained from anonymous healthy female donors during plastic surgery procedures according to the French regulations (article L.1243-4 of the French public Health Code) and Declaration of Helsinki act. Patients’ written informed consents were collected and kept by the surgeon. Only age, sex and anatomical site of samples were specified to the authors. The authors did not participate in sample collection. Given its special nature, surgical residue is subject to specific legislation included in the French Code of Public Health (anonymity, gratuity, sanitary/safety rules, etc.). This legislation does not require prior authorization by an ethics committee for sampling or use of surgical waste.

The full thickness human skins were stored at − 20 °C after surgery for less than 6 months before use. The skins were controlled visually after thawing; those with stretchmarks, holes, damages, etc., were discarded. Patches 32 mm in diameter were punched out from the skins. Skins were cleaned with pure water and remaining subcutaneous fat was removed. The skin thickness of each patch was measured using a micrometer; mean thickness was at 3107 ± 458 µm. Full thickness human skin was used instead of split thickness skin as it is easier to tape strip.

Each skin sample was set up on a static diffusion cell filled with phosphate buffer at 0.1 M/pH 7.4 with 2 cm^2^ exposure area. The receptor fluid was stirred during the entire treatment process. The skin was equilibrated for one hour, after which the skin temperature remained at 32 ± 1 °C, as measured using an infrared contactless probe. The skin integrity was checked by measuring trans-epidermal water loss (TEWL, Vapometer, Delfin, Westhumble, UK) and skin samples with TEWL values higher than 20 g m^-2^ h^-1^ were rejected. This cut off TEWL value was based on historical data obtained in our laboratory.

For the first study a solution (Propylene Glycol/Ethanol/Water 5/47.5/47.5 v/v/v) containing 3% JAD and 2% Caffeine was used. For the second study, the same vehicle was used with 2% Caffeine, 2% 2-MR and 1% Oxybenzone. In addition, the results presented in Supplementary Fig. S6 were obtained on a sample treated with a vehicle of Dipropylene Glycol/Water (2/1 w/w) containing 30% JAD as the only active.

The first study was done on a single donor in duplicate (only one skin sample was analyzed by 3D ToF SIMS). The second study was done on two different donors in duplicate (one sample per donor was analyzed by 3D ToF SIMS).

1 ml of the solution was applied onto the skin. The donor compartment was covered with aluminum foil to prevent vehicle evaporation. After 4 h, the formulation was removed and the skin surface was washed with a cotton tip soaked with gentle detergent solution at 10% in water, then rinsed with a second cotton tip soaked in water and dried with at least one additional cotton tip.

Twelve successive tape strip samples were prepared from each skin sample using D‐squame disks D100 (CuDerm Corporation, Dallas, TX, USA) with a diameter of 22 mm. Each individual strip was placed in a Petri dish, then shipped on dry ice and stored at − 20 °C until analysis (approximately 2 weeks). The second, fourth and tenth strips were shipped for 3D ToF–SIMS analysis. The remaining tape strips of the second study were analyzed for quantification of caffeine, 2-MR and oxybenzone by LC–MS/MS.

### Strip sample preparation and LC–MS/MS

Tape strips were extracted with 1 ml methanol with back and forth agitation at 300 rpm for 18 h. Caffeine, 2-MR and Oxybenzone was quantified on each strip with a validated analytical LC–MS/MS method (Supplementary Fig. S8).

### Scanning electron microscopy (SEM)

Tape strip samples from three of the donors were coated with 15 nm thick Au/Pd films and analysed using a Zeiss Supra 40VP FEG-SEM instrument at an electron energy of 2 keV and an Everhardt-Thornley type detector. SEM micrographs were acquired at the same areas that were previously analysed by 3D ToF–SIMS, as well as in areas not subjected to sputter erosion, displaying intact corneocytes. Superimposed ToF–SIMS and SEM images were obtained by manual alignment using Powerpoint (Microsoft, Inc.).

### Supplementary Information

Supplementary Figures.

## Data Availability

Data will be available upon reasonable request to the corresponding author.
