# Supplementary material for: Spatial distribution of active compounds in stratum corneum—partitioning between corneocytes and lipid matrix
Source: Sci Rep. 2024 Aug 12;14:18681. doi: 10.1038/s41598-024-66418-x (PMC11319811; doi:10.1038/s41598-024-66418-x)
Supplement: Supplementary file 1 — Supplementary Figures. [file 41598_2024_66418_MOESM1_ESM.pdf]

Supplementary information

Spatial distribution of active compounds in stratum corneum – partitioning between corneocytes and lipid matrix.

Peter Sjövall[1*], Sebastien Gregoire[2], William Wargniez[2], Lisa Skedung[3], Ann Detroyer[2] and Gustavo S. Luengo[2*]

[1] RISE Research Institutes of Sweden, Materials and Production, SE-50115 Borås, Sweden
[2] L'Oréal Research and Innovation, 93601, Aulnay-sous-Bois, France
[3] RISE Research Institutes of Sweden, Bioeconomy and Health, SE-11428 Stockholm, Sweden

* Corresponding authors: peter.sjovall@ri.se, gluengo@rd.loreal.com

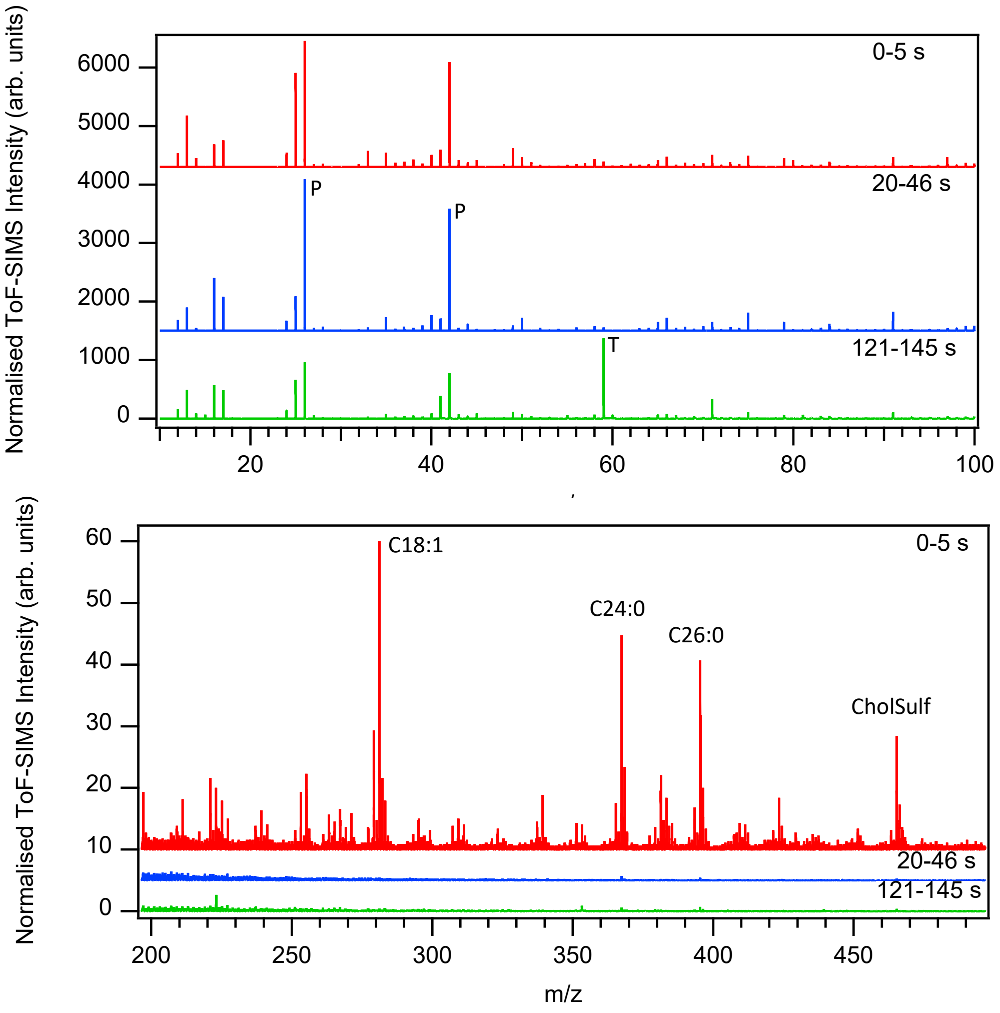

Supplementary Figure S1

Negative ion ToF-SIMS spectra from different depth intervals of the tape strip sample, representing the top lipid layer (0-5 s), corneocyte interior (20-46 s), and tape substrate with remaining protein residues (121-145 s). The spectra have been normalized to the duration of the time intervals, to allow for direct comparison of signal intensities between the spectra. Labels refer to peaks representing proteins (P), tape (T) and lipids (C18:1, C24:0, C26:0, CholSulf), see Table 1.

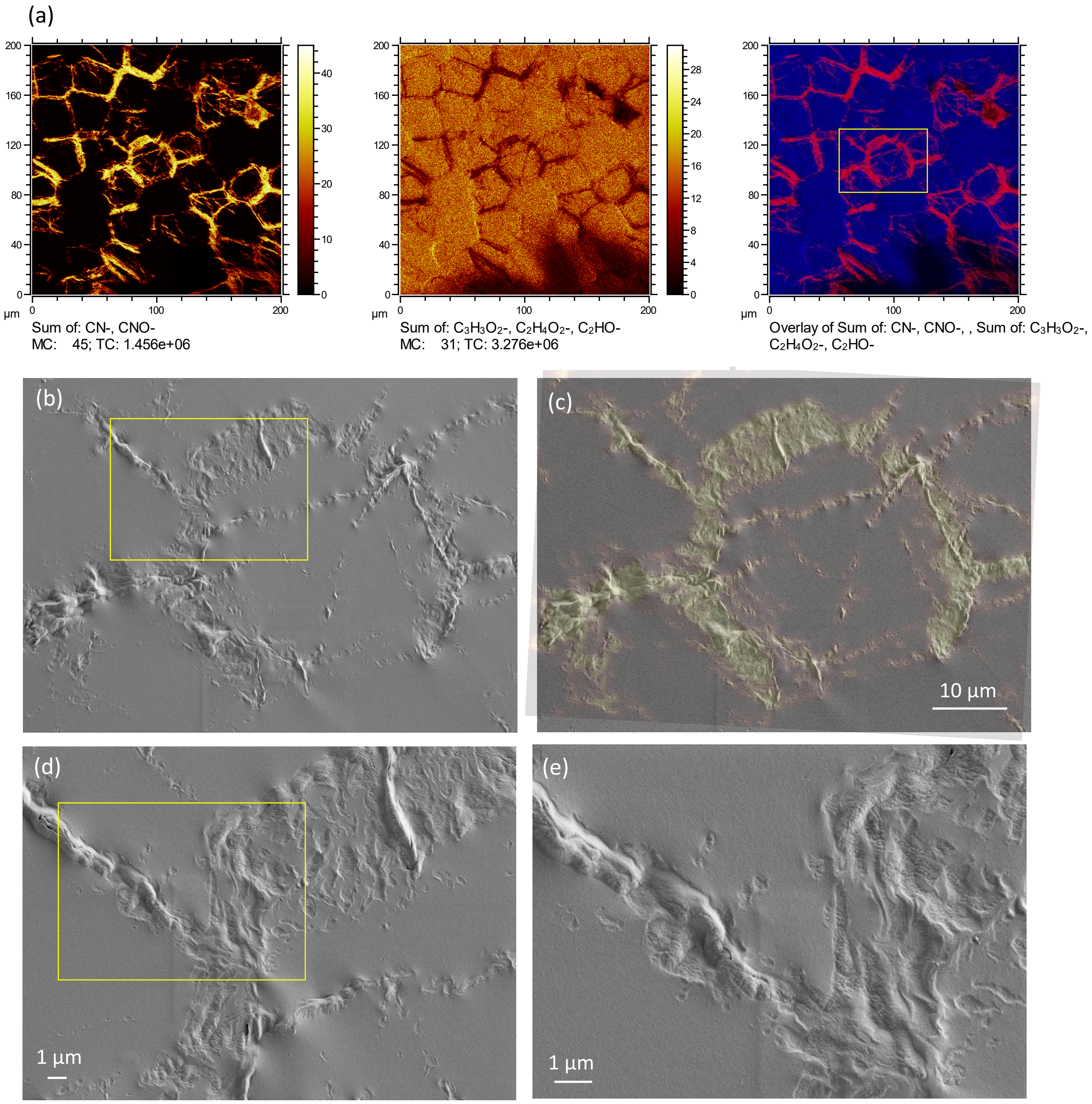

Supplementary Figure S2

ToF-SIMS and SEM images of tape strip sample (TS4) after 120 s sputter erosion. (a) Negative ion images of proteins and tape, respectively, and an overlay image of proteins (red) and tape (blue). (b) SEM micrograph and (c) protein ion image superimposed on SEM micrograph of area indicated by white frame in (a). (d) SEM micrograph of area indicated by yellow frame in (b), and (e) further magnified SEM micrograph of area indicated by yellow frame in (d).

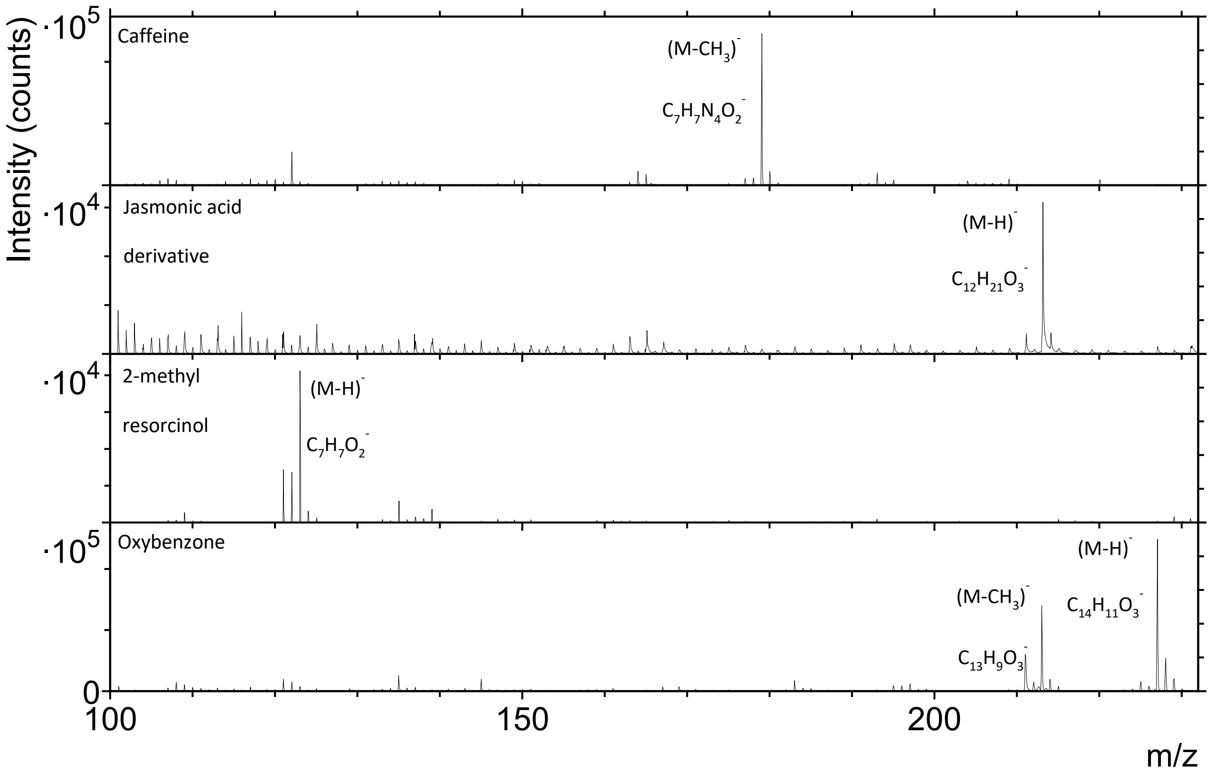

Supplementary Figure S3

Negative ion ToF-SIMS spectra of the four actives studied in this work.

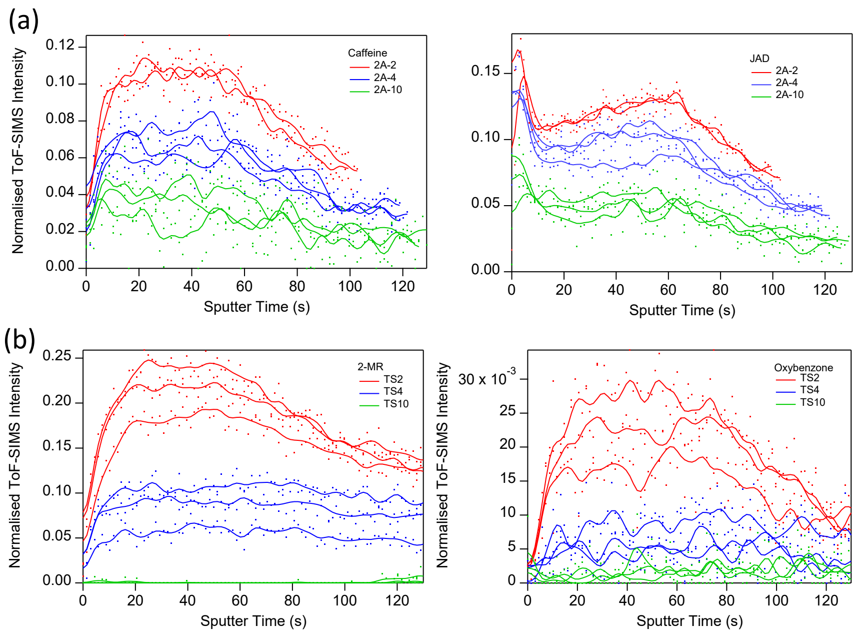

Supplementary Figure S4

Normalised depth profiles of actives in tape strip samples from skin treated with (a) a mixture of caffeine and JAD and (b) a mixture of caffeine, 2-MR and oxybenzone.

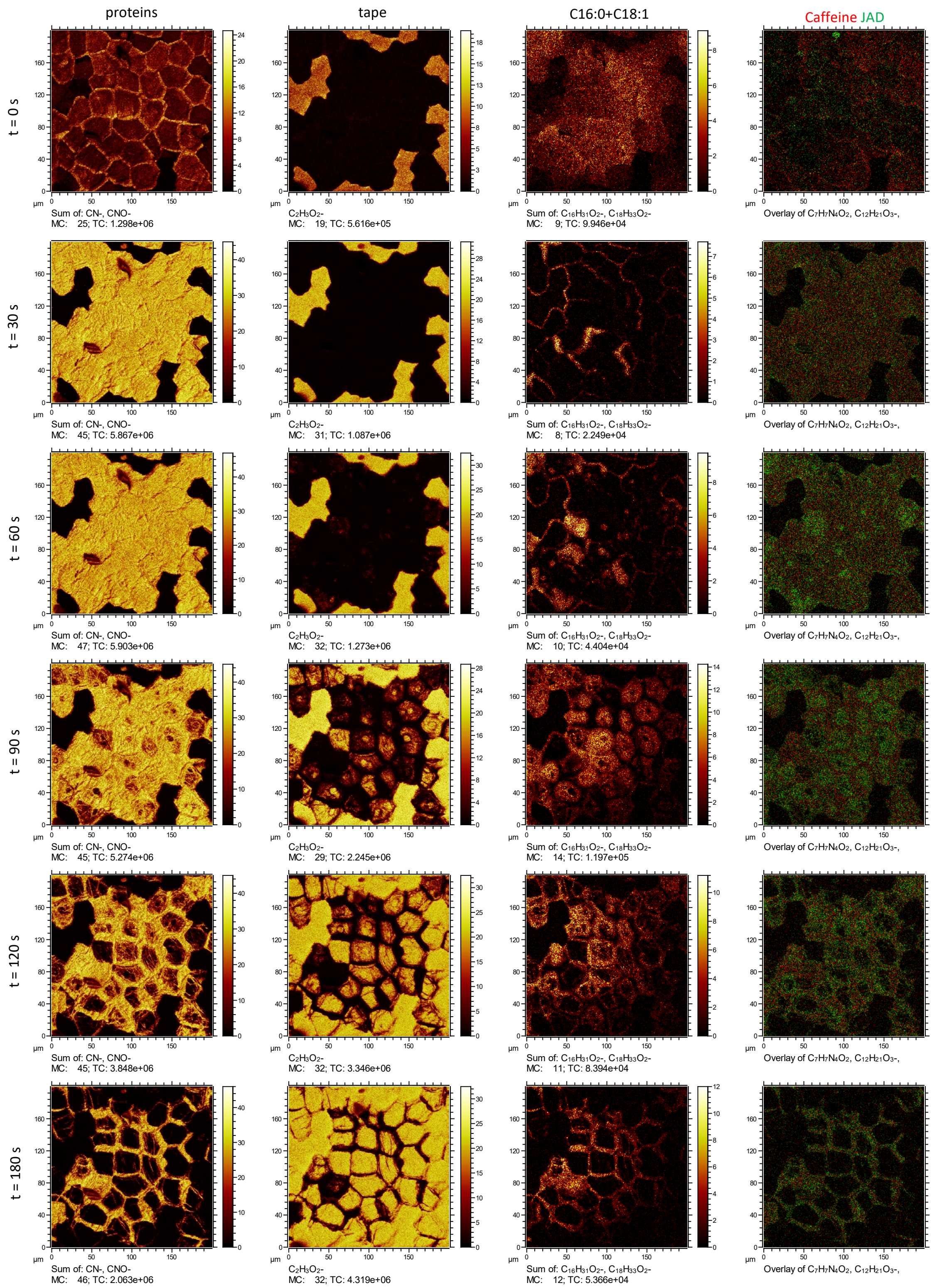

Supplementary Figure S5

Negative ion images of proteins, tape, C16:0+C18:1 and caffeine(red)/JAD(green) in tape strip (TS2, same measurement as in Fig. 3) from skin treated with a mixture of caffeine and JAD, after increasing sputter erosion times.

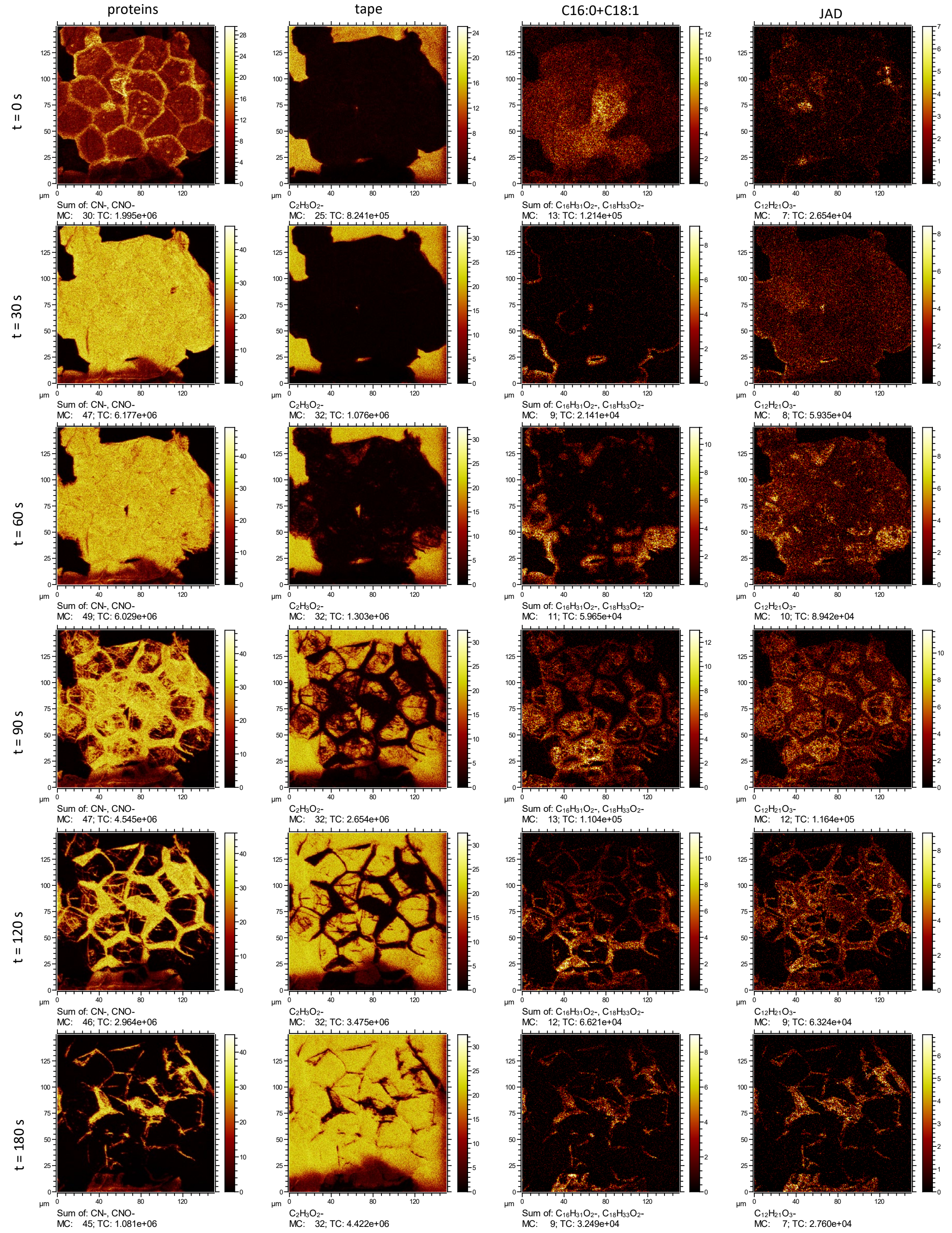

Supplementary Figure S6

Ion images of proteins, tape, C18:1 and JAD in tape strip (TS2) from skin treated with only JAD (high concentration, 30%), after increasing sputter erosion times.

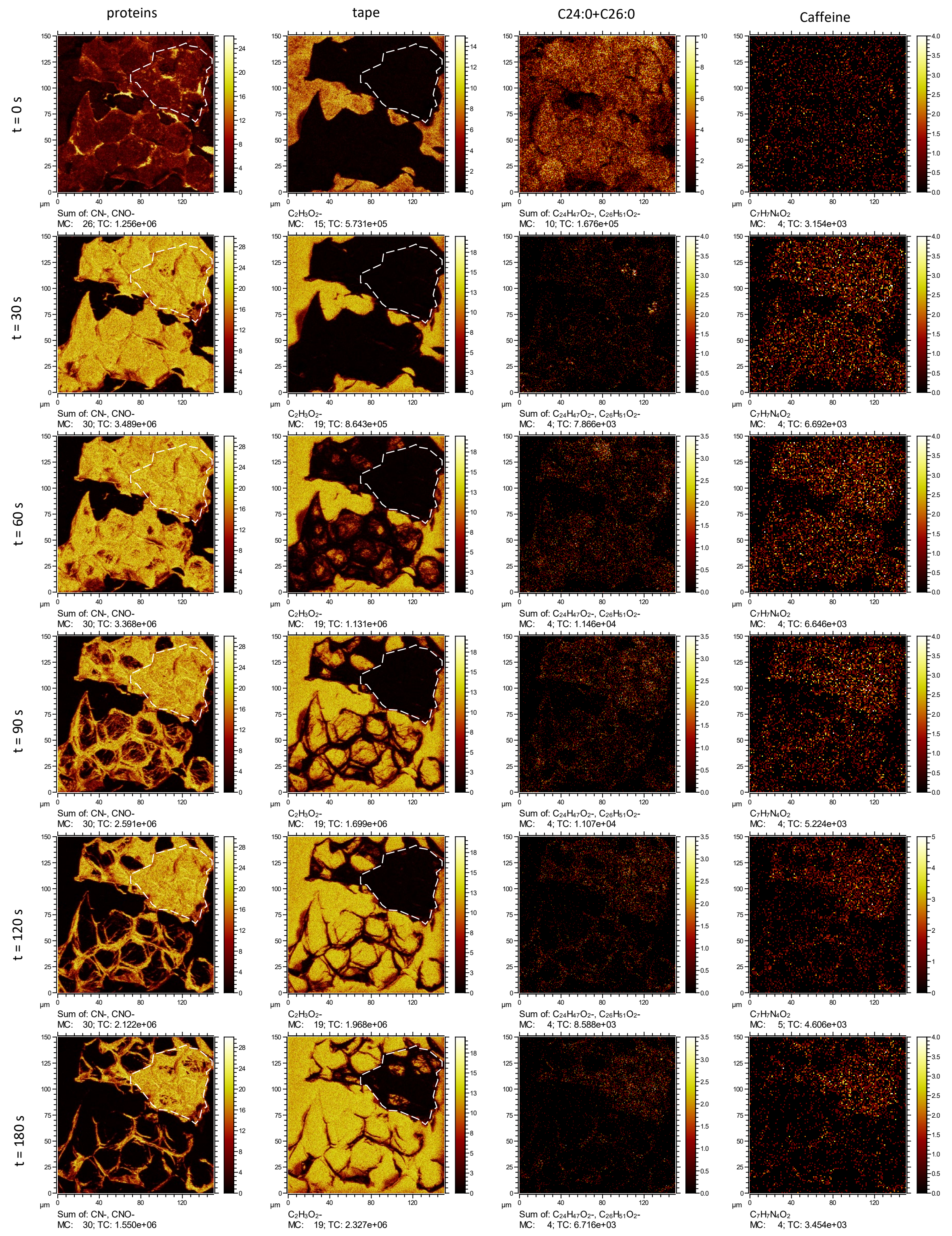

Supplementary Figure S7

Ion images of proteins, tape, C24:0+C26:0 and caffeine in tape strip (TS2) from skin treated with a mixture of caffeine, 2-MR and oxybenzone, after increasing sputter erosion times. An area attributed to corneocyte double layer is indicated by a dashed white line in the protein and tape images.

Analytical methods

Column	ascentis C18 50x2,1mm 2,7µm
Solvent A	Water + 0,1% formic acid
Solvent B	Acetonitril
Column temperature	50
Washing syringe	MeOH/water (50/50)
Injected solvent	MeOH/water (50/50)
Injected volume (µL)	5
Rinse dip time (sec)	5

Gradient	Time (min)	Flow rate (ml/nin)	A (%)	B (%)
	0.2	0.8	100	0
	1	0.8	0	100
	1.4	0.8	0	100
	1.41	0.8	100	0
	2	0.8	100	0

Products	Q1	Q3	Dwell	DP	EP	CE	CXP
Cafeine	195	138	150	80	7	24	10
Cafeine C13	198	140	150	80	7	24	10

Probe position	5 et 5	Gas1	45
Ionization mode	ESI	Gas2	45
Polarity	Pos	CAD	5
Acquisition mode	MRM	Nebulizer current	-
Resolution	unit	Curtain gas	25
Source temperature	600	Capilary voltage pos	5500
Interface temperature	100	Capilary voltage neg	/

DP : declustering potential (V)
EP : entrance potential (V)
CEP : Cell entrance potential (V)
CE : Colision energy (V)
CXP : cell exit poteltial (V)
Tr : retention time (min)

Caffeine

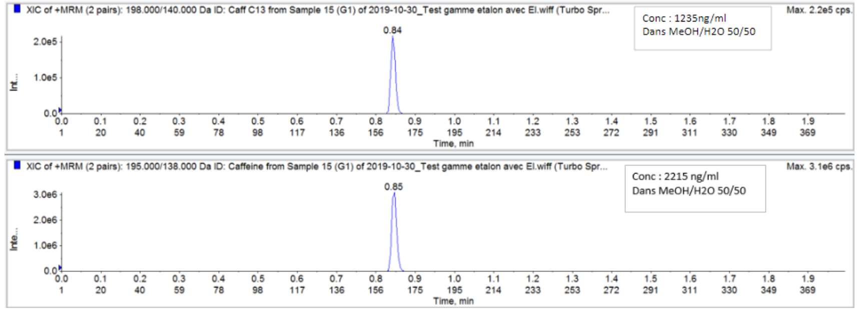

Supplementary Figure S8

Details of the LC-MS/MS analytical method used for caffeine, 2-MR and oxybenzone quantification in tape strip.
